# Electrochemical Immunosensor for Early Detection of β-Amyloid Alzheimer’s Disease Biomarker Based on Aligned Carbon Nanotubes Gold Nanocomposites

**DOI:** 10.3390/bios12111059

**Published:** 2022-11-21

**Authors:** Pushpesh Ranjan, Raju Khan

**Affiliations:** 1 CSIR—Advanced Materials and Processes Research Institute (AMPRI), Hoshangabad Road, Bhopal 462026, India; 2 Academy of Scientific and Innovative Research (AcSIR), Ghaziabad 201002, India

**Keywords:** Alzheimer’s disease, β-amyloid peptide, electrochemical immunosensor, gold nanoparticles, aligned carbon nanotube, chitosan

## Abstract

Beta-amyloid (βA) peptides accompanying the physiological change in brain induce Alzheimer’s disease. In this work, a highly sensitive electrochemical (EC) immunosensor platform has been developed for the quantitative detection of βA peptides, using the gold nanoparticle functionalized chitosan-aligned carbon nanotube (CS-aCNT-Au) nanocomposites on glassy carbon electrodes (GCE). The immunosensor has been fabricated by immobilization of the anti-βA antibody upon CS-aCNT-Au/GCE. In the CS-aCNT nanocomposite, CS has high biocompatibility. Hydroxy and amine functionalities favor the antibody immobilization and prevent the leaching of nanocomposites of the modified electrode due to the adhesive environment. Moreover, aCNT offers high conductivity, stability, and a large surface area (the calculated effective surface area of the CS-aCNT/GCE is 8.594 × 10^−2^ cm^2^). However, the incorporation of AuNPs further enhances the conductivity of the CS-aCNT-Au nanocomposite based on differential pulse voltammetry (DPV) results, and also improves the effective surface area (9.735 × 10^−2^ cm^2^). The surface morphology and electrochemical studies of the nanocomposite, as well as its modifications by the anti-βA antibody and BSA, were carried out through field emission scanning electron microscope (FESEM), cyclic voltammetry (CV), and DPV. The quantitative immunosensing of the βA in phosphate-buffered saline (PBS) solution is accomplished via DPV, which reveals that the immunosensor has a high sensitivity of 157.60 µA pg^−1^ mL cm^−2^ and a broad detection range of 10.0 pg mL^−1^–100.0 µg mL^−1^, with a limit of detection (LOD) of 0.87 pg mL^−1^. Subsequently, we detected the spiked βA in diluted serum with a linear detection range of 10.0 pg mL^−1^–1.0 ng mL^−1^ and LOD of 0.95 pg mL^−1^. Moreover, a selectivity study exhibited a high affinity of immunosensors towards βA. Thus, we propose that this highly efficient immunosensor can potentially be applied for the point-of-care (POC) sensing of βA in clinical samples.

## 1. Introduction

Alzheimer’s disease (AD) is a common neurological disorder in the human brain, which is paid high attention due to the results of dementia and millions of deaths. AD accounts for ~70% of dementia cases, of which ~35 million people have AD and this number is projected to expand by almost three times (~115 million) in 2050 [1,2,3]. AD is most common in the age group older than 65. However, approximately 6% of people have suffered from AD before the age of 65 years. The World Health Organization (WHO) estimates that AD causes a high global socio-economic burden of USD ~1.9 trillion by 2030 [4]. AD is the usual chronic and progressive form of neurodegenerative disease. Studies have revealed that AD is regulated through the growth of fibrils in the brain, known as amyloid peptides. One of their forms is the βA peptide, which is hydrophobic and self-aggregated in brain tissues, causing the primary incidence of AD. βA peptide is formed due to enzymatic degradation through β- and γ-secretase of βA protein from the amyloid precursor protein (APP). Therefore, the monomeric and aggregative form of βA is highly endorsed as a diagnostic biomarker and a therapeutic target. Thus, regular monitoring is essential to manage their progression and treatment [2,3,5,6].

To date, numerous diagnostic platforms, including capillary electrophoresis (CE), enzyme-linked immunosorbent assay (ELISA), magnetic resonance imaging (MRI), mass spectroscopy (MS), positron emission tomography (PET), and immunohistochemistry (IHC) have been employed for the detection of AD. However, the aforementioned methods are less sensitive, and also time-consuming, costly, and labor-intensive. Therefore, a highly selective, sensitive, and cost-effective detection technique is crucial for clinical practice for early diagnosis and therapeutic of AD [2,3,7].

Accordingly, the immunosensor-based diagnostic platform is considered the most promising tool for POC application. Numerous immunosensors, such as electrochemical, surface plasmon resonance (SPR), and surface-enhanced Raman spectroscopy (SERS), have been utilized for the detection of AD. Amongst, electrochemical immunosensor has attained much interest due to its remarkable advantages, including ease of use, cost-effectiveness, high sensitivity and selectivity, ultra-low detection limit, considerable stability, and rapid detection. Moreover, the electrochemical immunosensor is label-free and easy to fabricate, does not require pre-treatment of a clinical sample, requires a low sample volume, and offers an on-site testing facility. Due to the potential advantages, several attempts have been made to detect βA using an electrochemical immunosensor [8,9]. In a recent report, Devi et al. developed an Au-NiFe_2_O_4_-GO nanocomposite-based electrochemical immunosensor for βA biomarker detection [10]. However, Abbasi et al. detected the βA up to the pg mL^−1^ using a label-free electrochemical biosensor based on polymer-modified graphene [11]. Similarly, a polymeric nanoparticle-functionalized gold nanocomposites-based immunosensor has been designed by Zhao et al. to detect the βA early, up to a nanomolar concentration [12].

Owing to fascinating physical and chemical properties, aCNT paves the way for highly efficient material in immunosensor design and fabrication. They have several advantages, including high surface area, light weight, high electrical conductivity, chemical stability, and excellent electrochemical properties. Moreover, the aligned geometry acts as a molecular wire, which offers high electron transfer, resulting in a highly sensitive immunosensor [13,14,15,16]. Nevertheless, chitosan (CS) has amine- and hydroxy-functional groups that offer active sites for the immobilization of antibodies in immunosensor fabrication. Additionally, the strong adhesive character makes a uniform coating of nanocomposite on the electrode surface and prevents the leaching of materials, which provides a stable environment for the fabrication of immunosensors [17]. Moreover, the functionalization of AuNPs remarkably improved the conductivity and effective surface area of the immunosensor. Thus, they effectively improved the sensitivity of the immunosensor [18,19].

Herein, we report a sensitive electrochemical immunosensor for βA detection. Firstly, we synthesized the AuNP-functionalized CS-aCNT nanocomposite, in which aCNT has high electroconductivity and surface area. However, the presence of amine and oxygen functionalities on CS favors antibody immobilization on the electrode surface, and the adhesive behavior of CS prevents the leaching of the nanocomposite. Additionally, AuNPs further improved the working efficiency of the immunosensor through the enhancement of conductivity and effective surface area of the nanocomposite. Afterward, the surface morphology of the CS-aCNT-Au nanocomposite and its successful modification by anti-βA antibodies and BSA has been studied by FESEM. Subsequently, we fabricated the working electrode by the surface modification of GCE using CS-aCNT-Au, followed by immobilization of anti-βA antibodies and BSA. Further, the electrochemical characteristics of the nanocomposites and immunosensor have been studied through electroanalytical techniques, such as CV and DPV. The fabricated immunosensor has been used for the detection of the varied concentration of βA in PBS and the achievement of a LOD of 0.87 pg mL^−1^. We tested the immunosensor performance in diluted serum as well as selectivity, where it showed remarkable results concerning βA detection.

## 2. Experimental Details

### 2.1. Chemicals

The monoclonal βA antibodies and βA peptides were purchased from Abcam, UK. Aligned carbon nanotubes (aCNT) were purchased from TCI chemicals, India. Chitosan, gold (III) chloride trihydrate, sodium phosphate dibasic dihydrate, sodium chloride, potassium chloride, sodium phosphate monobasic dihydrate, bovine serum albumin, potassium ferrocyanide, potassium ferricyanide, *N*-hydroxysulfosuccinimide sodium (NHS), *N*-(3-Dimethylaminopropyl)-*N*’-ethyl carbodiimide hydrochloride (EDC), dopamine, immunoglobulin G, and cortisol were procured from Sigma-Aldrich, USA. The deionized (DI) water was obtained from the Millipore system.

### 2.2. Preparation of Chitosan and Chitosan-Aligned Carbon Nanotube

In order to prepare the chitosan solution, a 500 mg chitosan flake was mixed with the 10 mL solution of 0.05 M acetate buffer. Then, the solution was constantly stirred overnight at 25 °C to make a transparent solution of CS. Moreover, the CS-aCNT nanocomposite has been prepared by mixing 5.0 mg aCNT with 1.0 mL of prepared CS solution. The solution was bath-sonicated at 1 h and then constantly stirred for 1 h at 25 °C to perform the uniform mixing of aCNT into the CS solution [17,20].

### 2.3. Preparation of Gold Nanoparticles

The AuNPs were synthesized chemically from auric chloride salt through the reduction of citrate salt. In detail, a 0.5 mM of 100 mL HAuCl_4_·3H_2_O was refluxed at 60 °C for 20 min with continuous stirring. Then, 50 mL of trisodium citrate (50 mM) was added dropwise into gold aqueous solution, where the color of the solution appeared red, denoting the formation of AuNPs. When the red color of the AuNPs solution appeared, it was further refluxed for 20 min and cooled at room temperature with continuous stirring. The prepared AuNPs solution was stored at 4 °C for future applications [21].

## 3. Result and Discussions

### 3.1. Physical Characterizations

**Figure 1:**
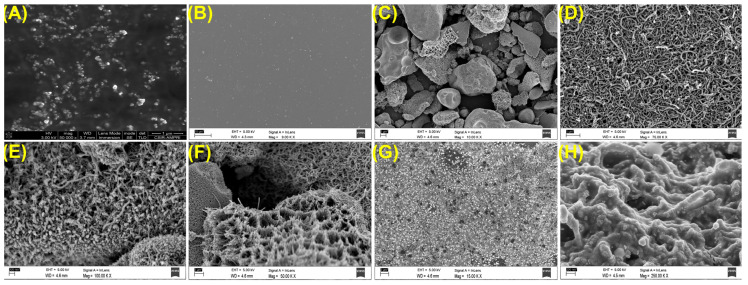
FESEM images of (**A**) AuNPs, (**B**) CS, (**C**,**D**) CS-aCNT nanocomposite, (**E**,**F**) CS-aCNT-Au nanocomposite, (**G**) antibody/CS-aCNT-Au nanocomposite, and (**H**) BSA/antibody/CS-aCNT-Au nanocomposite.

The FESEM (Make: Carl ZEISS Microscopy, ZEISS Sigma, Germany) images of the prepared nanocomposites illustrated their successful synthesis [22]. The AuNPs, with a spherical size and uniform distribution, are shown in Figure 1A. However, a thick layer of CS film had a rough surface with several tiny particles which is displayed in Figure 1B. On the other hand, Figure 1C,D CS-aCNT nanocomposite illustrated the incorporation of aCNT on the CS surface as well as the stacking of the nanocomposite. The stacking may be due to the contraction of the nanocomposite after drying [23]. However, the AuNP-modified CS-aCNT nanocomposite is shown in Figure 1E,F. It has been pictured that the AuNPs embedded on the CS-aCNT surface designate the effective attachment of AuNPs in the CS-aCNT-Au nanocomposite [24]. However, the immobilization of anti-βA antibodies and BSA onto the CS-aCNT-Au nanocomposite surface is shown in Figure 1G,H, respectively. It should be noted that the surface of the CS-aCNT-Au nanocomposite is completely covered by a thick layer of antibodies and BSA. The results revealed the successful immobilization of anti-βA antibodies and BSA on the nanocomposite.

### 3.2. Fabrication of the Working Electrode

The GCE surface was modified for the fabrication of an electrochemical working electrode through the drop-casting method. Before the modifications of the GCE, it was hand-polished using alumina slurry (0.3 and 0.05 µM), and then cleaned through ethanol and DI water to eliminate the alumina particles from the electrode surface and to obtain a mirror-like surface [25]. Subsequently, a 5.0 µL CS-aCNT nanocomposite was drop cast onto the GCE and dried at atmospheric conditions. Afterward, 5.0 µL AuNPs solution was drop cast onto the modified surface and dried in a similar conditions to fabricate CS-aCNT-Au/GCE. Correspondingly, CS-aCNT/GCE was fabricated using the aforementioned method. Then, the fabricated working electrode was studied electrochemically via CV and DPV techniques.

### 3.3. Fabrication of the Immunosensor

**Scheme 1:**
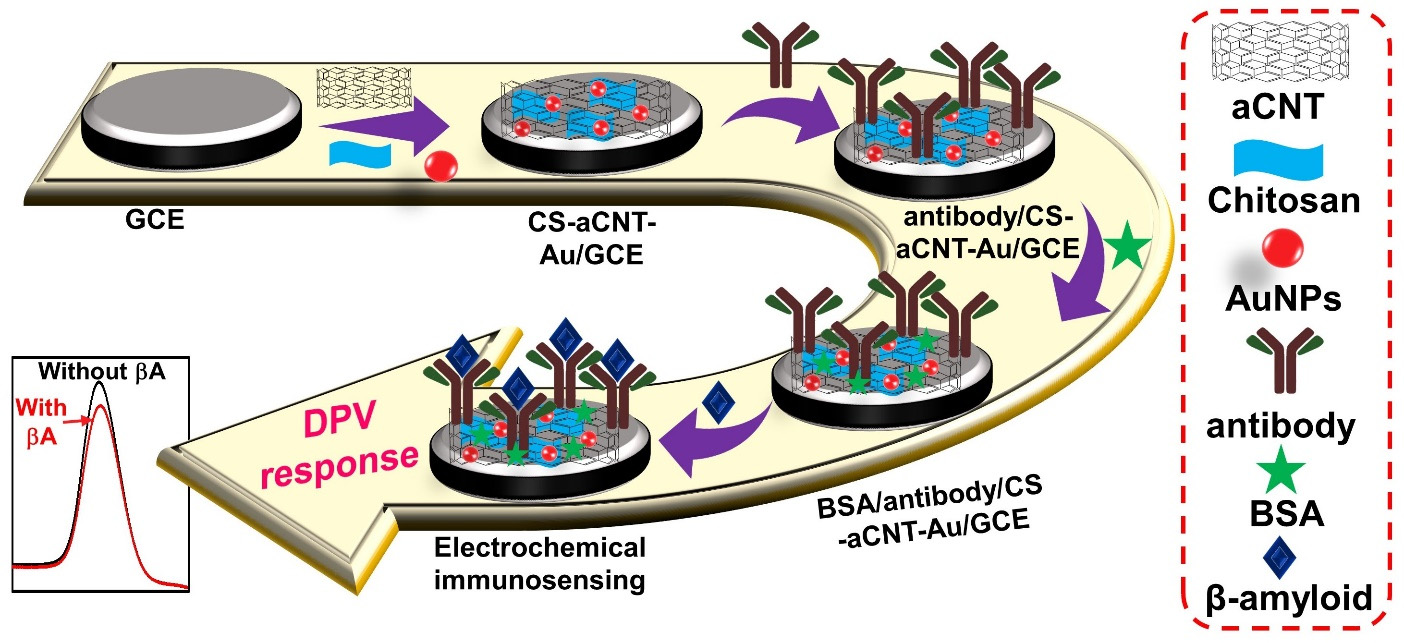
Schematic of the fabrication of the BSA/antibody/CS-aCNT-Au immunosensor.

The electrochemical immunosensor was fabricated by the modification of CS-aCNT-AuNPs/GCE. In detail, 5.0 µL of (4:1) EDC/NHS was drop cast onto a CS-aCNT-Au/GCE surface, where EDC/NHS functioned as a coupling mediator between the nanocomposite and biomolecules, i.e., antibodies, to allow covalent immobilization on the electrode surface. However, the unbounded EDC/NHS on the electrode surface was eliminated through washing with DI water. Subsequently, 5.0 µL of 10.0 µg mL^−1^ anti-βA antibody solution was immobilized on a modified CS-aCNT-Au/GCE surface and kept overnight at 4 °C. Afterward, it was washed using phosphate buffered solution (pH = 7.0) to eliminate the unbonded antibody. Furthermore, we immobilized the 1% bovine serum albumin (BSA) on the antibody modified electrode surface for an hour, which was further washed to remove the unnecessary BSA. Here, BSA acted as a blocking agent that blocked the reactive surface of the electrode, which left after antibody immobilization. The blocking is necessary to prevent the non-specific responses of the modified electrode; otherwise, it would cause the incorrect results. After the complete fabrication of the immunosensor, which is termed a “BSA/antibody/CS-aCNT-AuNPs/GCE”, it was stored at 4 °C for electrochemical study. A schematic illustration of the stepwise modifications of the immunosensor is displayed in Scheme 1.

## 4. Electrochemical Studies of the Modified Electrodes

**Figure 2:**
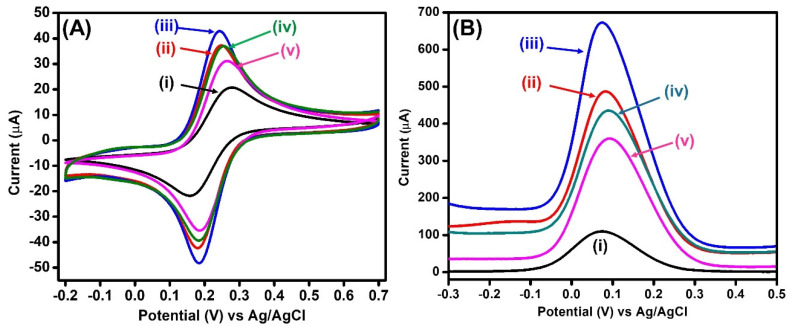
(**A**) CV and (**B**) DPV measurements of (i) GCE, (ii) CS-aCNT/GCE, (iii) CS-aCNT-Au/GCE, (iv) antibody/CS-aCNT-Au/GCE, and (v) BSA/antibody/CS-aCNT-Au/GCE.

The studies on the electrochemical properties of the fabricated electrodes were conducted through electroanalytical techniques such as CV and DPV [26,27]. All of the electrochemical measurements were performed on a Metrohm P/G (Autolab) EC workstation (Model-PGSTAT204, Netherland) using the 3-electrodes system (fabricated working electrode, an Ag/AgCl reference electrode and a Pt-wire counter electrode) in PBS, pH = 7.4, containing 0.9% KCl and 5 mM ferro/ferricyanide [Fe(CN)_6_]^−3/−4^ redox chemical in a −0.2 to 0.7 V potential range at a scan rate of 0.015 Vs^−1^. As demonstrated in Figure 2A, the anodic peak current (Ipa) of the GCE was calculated to be 24.012 µA, at an anodic peak potential (Epa) of 0.276 V. However, after the GCE modifications, the Ipa of CS-aCNT/GCE and CS-aCNT-Au/GCE increased, and was calculated of 37.994 and 43.036 µA at an Epa of 0.247 and 0.243 V, respectively. It should be noted that the incorporation of AuNPs remarkably enhanced the Ipa of CS-aCNT-Au nanocomposites, suggesting that the AuNPs have a high conductive nature. CS-aCNT-Au/GCE displayed the highest Ipa amongst the other modified electrodes. Nevertheless, with the immobilization of the anti-βA antibody (antibody/CS-aCNT-Au/GCE), followed by the BSA blocking agent (BSA/antibody/CS-aCNT-Au/GCE), the Ipa reduced to 37.780 and 34.710 µA, at Epa of 0.254 and 0.265 V, respectively. The reduced CV peak currents suggested effective immobilization of anti-βA antibody and BSA on the electrode surface, which could potentially detect the βA.

Moreover, we also calculated the effective surface area of the fabricated electrodes using the Randal–Sevcik equation (Equation (1)) [19]:

Ip = (2.69 × 10^5^) An^3/2^Cυ^1/2^D^1/2^ (1)

where Ip and A represent the peak current and the effective surface area (A**_effective_**) of the modified electrodes, respectively. n represents electron(s) participating in the electrochemical process (in this case, n = 1), C is the concentration of the redox chemical (in this case, 5 mmol cm^−3^), υ represents the scan rate (0.015 Vs^−1^), and D denotes the diffusion coefficient (7.26 × 10^−6^ cm^2^ s^−1^) [19].

The A of the fabricated electrodes was directly proportional to the resulting peak current of the electrochemical system, where the A of the GCE was calculated to be 5.433 × 10^−2^ cm^2^. However, for CS-aCNT/GCE and Cs-aCNT-Au/GCE, the A increased to 8.594 × 10^−2^ cm^2^ and 9.735 × 10^−2^ cm^2^, respectively. The enhancement of the A of the modified electrodes indicated the improved conductivity and high surface area of aCNT and AuNPs. Nonetheless, A slightly reduced to 8.546 × 10^−2^ cm^2^ and 7.854 × 10^−2^ cm^2^ for antibody/CS-aCNT-Au/GCE and BSA/antibody/CS-aCNT-Au/GCE, respectively. This reduction marked the reduced conductivity of the modified electrode, which was achieved after the successful immobilization of the anti-βA antibody and BSA on the CS-aCNT-Au/GCE surface.

Moreover, the Brown–Anson model was employed to calculate the surface concentration on the antibody/CS-aCNT-Au/GCE bioelectrode using Equation (2) [10].

(2) $$\mathrm{Ip}=\frac{n^{2}F^{2}I^{*}A\upsilon }{4\mathrm{RT}}$$

In this equation, n denotes the number of electrons involved in the reaction (n = 1), F represents the Faraday constant (96,485 C mol^−1^), I* is the surface concentration of redox chemical (mol cm^−2^), A is the effective surface area of the working electrode, υ is the scan rate (Vs^−1^), R is the universal gas constant (8.314 J mol^−1^ k^−1^), and T is the absolute temperature (300 K). The surface concentration of the redox chemical on the antibody/CS-aCNT-Au/GCE bioelectrode was calculated to be 3.52 × 10^−8^ mol cm^−2^.

At the same time, the DPV technique was performed in order to evaluate the property of the modified electrodes by measuring the peak current. As displayed in Figure 2B, the DPV peak current was measured at 109.915 µA for GCE. However, after the modification of the CS-aCNT and CS-aCNT-Au nanocomposite, it remarkably increased to 487.0 and 672.609 µA, respectively. The enhanced peak current also represents the high conductive nature of the nanocomposite, as well as the successful modification of the GCE surface. Furthermore, the peak current decreases to 435.356 and 359.743 µA after the immobilization of the anti-βA antibody, succeeded by the BSA on CS-aCNT-Au/GCE. The reduction in the peak current revealed the effective immobilization of anti-βA antibodies and BSA, where the bulkiness of biospecies obstructed the flow of electrons in redox PBS solution and caused low conductivity. The DPV results are consistent with the CV measurements of modified electrodes. Therefore, we conclude that the fabricated immunosensor can efficiently work for the electrochemical detection of βA.

### 4.1. Scan Rate Study

**Figure 3:**
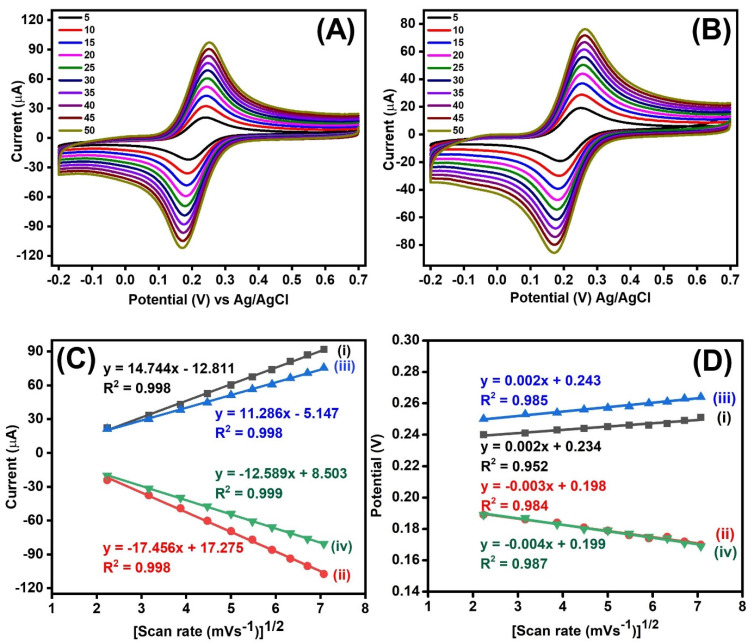
Studies of the scan rate effect of (**A**) CS-aCNT-Au/GCE, (**B**) BSA/antibody/CS-aCNT-Au/GCE. The respective straight-line calibration curve of (**C**) the peak current, (i) Ipa and (ii) Ipc of CS-aCNT-Au/GCE, (iii) Ipa and (iv) Ipc of BSA/antibody/CS-aCNT-Au/GCE, and (**D**) the peak potential, (i) Epa and (ii) Epc of CS-aCNT-Au/GCE, (iii) Epa and (iv) Epc of BSA/antibody/CS-aCNT-Au/GCE.

The electrochemical properties, including electrode reversibility; redox properties, such as oxidation and reduction in peak current and peak potential of anodes and cathodes; and kinetics of the electroactive materials, were studied through the CV at varying scan rates [28,29]. The redox kinetics of the CS-aCNT-Au/GCE and BSA/antibody/CS-aCNT-Au/GCE immunosensors studied in PBS, containing ferro/ferricyanide redox chemicals, had varying scan rates, from 0.005 to 0.050 Vs^−1^ at the interval of 0.005 Vs^−1^. As illustrated in Figure 3A,B, the Ipa of both electrodes gradually increased with the function of the scan rate. However, the cathodic peak current (Ipc) decreased similarly to the scan rate. The straight-line correlation of the square root of the scan rate vs. Ipa and Ipc calculated high correlation coefficients (R^2^) of 0.998 and 0.998 and 0.998 and 0.999 for CS-aCNT-Au/GCE and BSA/antibody/CS-aCNT-Au/GCE, respectively (Figure 3C). The results revealed excellent diffusion-controlled electron transfer kinetics of both electrodes, and stated the quasi-reversible characteristics. Nevertheless, the Epa and cathodic peak potential (Epc) slightly increased and decreased with the varying scan rate (Figure 3D).

### 4.2. Quantitative Analysis of β-Amyloid Peptide

**Figure 4:**
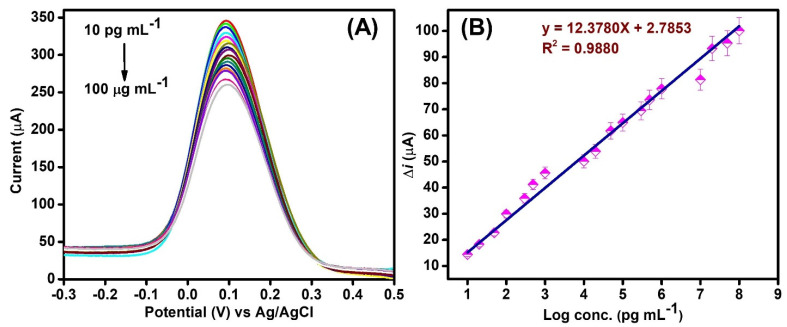
Immunosensor performance for βA in PBS. (**A**) The voltammetric response of the BSA/antibody/CS-aCNT-Au/GCE for the βA from 10.0 pg mL^−1^ to 100.0 µg mL^−1^. (**B**) The related calibration curve of the immunosensor.

The quantitative estimation of the βA has been carried out using the BSA/antibody/CS-aCNT-Au/GCE immunosensor via the DPV technique. In the DPV, the peak height is proportional to the concentration of the target analyte. Moreover, DPV takes a short time to generate the electrochemical signal. Therefore, DPV uses a more suitable technique for rapid quantification of the analyte of interest [30]. Herein, we analyzed the broad concentrations of βA, ranging from 10.0 pg mL^−1^ to 100.0 µg mL^−1^, in PBS (Figure 4A). We experimentally detected that the DPV current gradually decreases with the increase in βA concentration. The appearance of a reduced peak current is attributed to the bulkiness of the electrode surface after the binding of βA with the anti-βA antibody of the electrode surface. A bulkier surface would retard the electron flow, resulting in a low peak current response. The LOD and limit of quantification (LOQ) of the immunosensor were calculated using Equations (3) and (4), respectively [31].

(3) $$\mathrm{LOD}=\frac{3s}{m}$$

(4) $$\mathrm{LOQ}=\frac{10s}{m}$$

where S is the standard deviation (SD) [SD = S.E. × √N], N denotes the number of samples, and S.E. and m represent the standard error of the intercept and slope, respectively.

The measured quantitative values of the LOD and LOQ of the immunosensor were 0.87 pg mL^−1^ and 2.95 pg mL^−1^, respectively. In addition, the immunosensor had a high sensitivity of 157.60 µA pg^−1^ mL cm^−2^, which is estimated by using the formula for the sensitivity = S/A, where S and A are the slope of the calibration graph and the effective surface area of the immunosensor, respectively.

Additionally, the high R^2^ value (0.988) revealed the excellent performance of the immunosensor in wide-ranging concentrations (Figure 4B). Therefore, we determined that the immunosensor has a high potential that can efficiently detect much lower concentrations, up to the picogram level for βA, as well as having a wide detection range and high sensitivity. A comparative analytical performance of the previously reported immunosensor with our developed immunosensor for βA detection is listed in Table 1.

### 4.3. Detection of β-Amyloid Peptide in Biological Fluids

**Figure 5:**
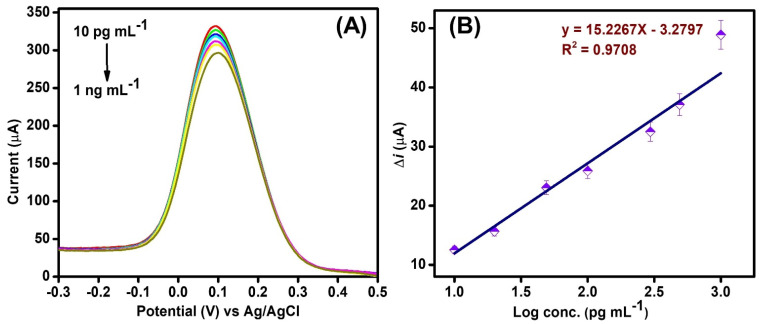
Immunosensor performance for βA in biological fluids. (**A**) The voltammetric response of the BSA/antibody/CS-aCNT-Au/GCE for the βA from 10.0 pg mL^−1^ to 1.0 ng mL^−1^. (**B**) The related calibration curve of the immunosensor.

The constructed BSA/antibody/CS-aCNT-Au/GCE immunosensor was employed for the detection of spiked βA in diluted serum via the DPV technique in order to validate the clinical applicability. Before the analysis, we prepared the diluted serum samples by mixing a 1:10 volume ratio of serum with PBS. Afterward, it spiked with βA of varied known concentrations, from 10.0 pg mL^−1^ to 1.0 ng mL^−1^. It was observed that the electrochemical results in diluted serum samples showed consistency, as was detected in PBS (Figure 5A). The calculated LOD and LOQ of the immunosensor were 0.95 and 3.17 pg mL^−1^, with an R^2^ of 0.970 (Figure 5B). Therefore, the results revealed that the proposed method could efficiently detect the βA in the biological sample, and could act as an alternative to traditional detection methods.

### 4.4. Assessment of Selectivity of the Immunosensor

**Figure 6:**
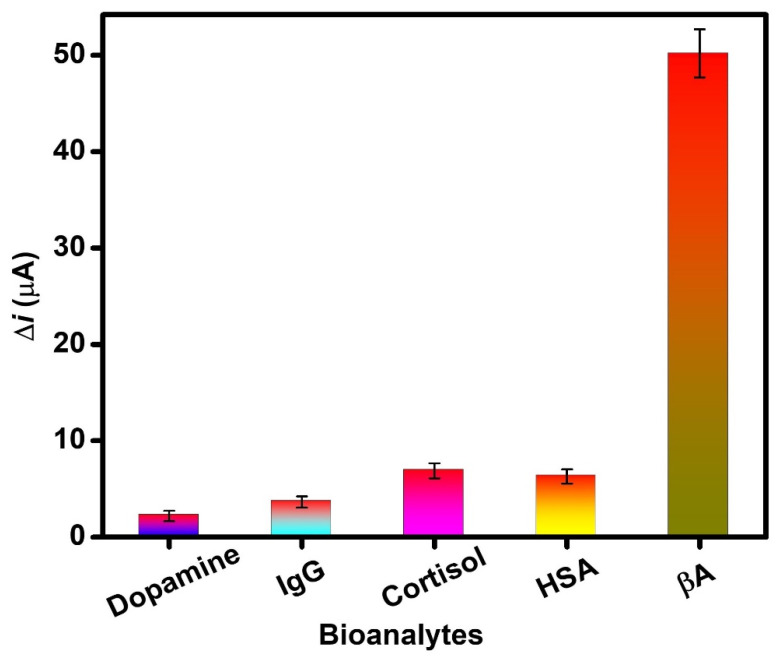
Selectivity of the immunosensor against various bioanalytes.

In order to examine the selectivity of the proposed immunosensor towards βA, it was tested against different bioanalytes, such as dopamine (10.0 ng mL^−1^), immunoglobulin G (IgG) (10.0 ng mL^−1^), cortisol (10.0 ng mL^−1^), and human albumin serum (HSA) (10.0 ng mL^−1^). It was shown that under similar experimental conditions, the change in current (Δ*i*) for other bioanalytes was very low compared to βA (10.0 ng mL^−1^) (Figure 6). The much smaller change in current revealed the negligible interference, which demonstrated the high selectivity of the immunosensor. Hence, it can be concluded that the proposed immunosensor selectively detects βA.

## 5. Conclusions

In conclusion, a highly sensitive and efficient electrochemical immunosensor has been developed for βA detection. A CS-aCNT-Au nanocomposite was utilized for fabrication of the working electrode. aCNT provides high conductivity, large surface area, and stability, while chitosan prevents the leaching of the nanocomposite and favors antibody immobilization. Moreover, the functionalization of AuNPs remarkably improved the conductivity and effective surface area, as examined through the CV and DPV measurements. The BSA/antibody/CS-aCNT-Au/GCE immunosensor showed high sensitivity for the detection of a wide range of concentrations of βA, from 10.0 pg mL^−1^ to 100.0 µg mL^−1^, with a LOD of 0.87 pg mL^−1^. Moreover, the consistent results of detection of βA in biological fluid, similarly to PBS, as well as the considerable selectivity of the immunosensor, showed a high ability to detect βA. Thus, the CS-aCNT-Au nanocomposite-based immunosensor exhibits remarkable potential for early diagnosis of βA against AD, which may be able to pave the way for timely treatment and disease management.

## Figures and Tables

**Table 1:** Comparative analytical performance of the immunosensor for βA detection.

Material	Technique	Linear Detection Range	LOD	Ref.
Au/NiFe_2_O_4_@GO-Ch/GCE	DPV	1.0 pg mL^−1^–1.0 ng mL^−1^	3.0 pg mL^−1^	[10]
Graphene/SPE	DPV	1.0 pg mL^−1^–1000.0 pg mL^−1^	1.4 pg mL^−1^	[11]
MIP/CI-HME	SWV	0.1 ng mL^−1^–1.0 μg mL^−1^	67.0 pg mL^−1^	[32]
CS-aCNT-Au/GCE	DPV	10.0 pg mL^−1^–100.0 µg mL^−1^	0.87 pg mL^−1^	This work

SWV = square-wave voltammetry.
